# Near-death experiences after cardiac arrest: a scoping review

**DOI:** 10.1007/s44192-024-00072-7

**Published:** 2024-05-28

**Authors:** Joshua G. Kovoor, Sanjana Santhosh, Brandon Stretton, Sheryn Tan, Hasti Gouldooz, Sylviya Moorthy, James Pietris, Christopher Hannemann, Long Kiu Yu, Rhys Johnson, Benjamin A. Reddi, Aashray K. Gupta, Morganne Wagner, Gregory J. Page, Pramesh Kovoor, Tarun Bastiampillai, Ian Maddocks, Seth W. Perry, Ma-Li Wong, Julio Licinio, Stephen Bacchi

**Affiliations:** 1 University of Adelaide, Adelaide, South Australia Australia; 2 Ballarat Base Hospital, Ballarat, VIC Australia; 3 Royal Adelaide Hospital, Adelaide, South Australia Australia; 4 Health and Information, Adelaide, South Australia Australia; 5 Heart of the Nation, Sydney, NSW Australia; 6 University of Queensland, Brisbane, QLD Australia; 7 Gold Coast University Hospital, Gold Coast, QLD Australia; 8 Bond University, Gold Coast, QLD Australia; 9 State University of New York Upstate Medical University, Syracuse, NY USA; 10 Westmead Hospital, Sydney, NSW Australia; 11 University of Sydney, Sydney, NSW Australia; 12 Flinders University, Adelaide, South Australia Australia; 13 Flinders Medical Centre, Adelaide, South Australia Australia; 14 Australasian Chapter of Palliative Medicine, Royal Australasian College of Physicians, Adelaide, South Australia Australia; 15 Lyell McEwin Hospital, Adelaide, South Australia Australia

## Abstract

**Background:**

This scoping review aimed to characterise near-death experiences in the setting of cardiac arrest, a phenomenon that is poorly understood and may have clinical consequences.

**Method:**

PubMed/MEDLINE was searched to 23 July 2023 for prospective studies describing near-death experiences in cardiac arrest. PRISMA-ScR guidelines were adhered to. Qualitative and quantitative data were synthesised. Meta-analysis was precluded due to data heterogeneity.

**Results:**

60 records were identified, of which 11 studies involving interviews were included from various countries. Sample size ranged from 28–344, and proportion of female patients (when reported) was 0–50%, with mean age (when reported) ranging 54–64 years. Comorbidities and reasons for cardiac arrest were heterogeneously reported. Incidence of near-death experiences in the included studies varied from 6.3% to 39.3%; with variation between in-hospital (6.3–39.3%) versus out-of-hospital (18.9–21.2%) cardiac arrest. Individual variables regarding patient characteristics demonstrated statistically significant association with propensity for near-death experiences. Reported content of near-death experiences tended to reflect the language of the questionnaires used, rather than the true language used by individual study participants. Three studies conducted follow-up, and all suggested a positive life attitude change, however one found significantly higher 30-day all-cause mortality in patients with near-death experiences versus those without, in non-controlled analysis.

**Conclusions:**

From prospective studies that have investigated the phenomenon, near-death experiences may occur in as frequent as over one-third of patients with cardiac arrest. Lasting effects may follow these events, however these could also be confounded by clinical characteristics.

**Supplementary Information:**

The online version contains supplementary material available at 10.1007/s44192-024-00072-7.

## Introduction

Near-death experiences are unusual phenomena that may occur at times of mortal peril. These experiences may include profound feelings of transcendence. Those who have had a near-death experience have been described to, at times, have longstanding and pervasive changes in outlook and behaviour. Despite decades of research and scientific discourse on the subject, the frequency, causes, and consequences of near-death experiences remain incompletely understood [1]. Near-death experiences can occur in various conscious states, and are not limited to those who have undergone cardiac arrest (clinical death) followed by resuscitation [2]. However, when scientifically evaluating the topic, the most consistent circumstances relate to cardiac arrest. The consistent description and evaluation of near-death experiences is also central to research on the topic. Prominent tools such as the Near-Death Experience scale developed by Greyson, that provides a validated method of distinguishing experiencers and non-experiencers [3], and the life-change inventory by Ring that seeks to characterise meaning within near-death experiences [4], have value and are used, however also have individual major limitations. Notably, the concluding themes in many of the studies using these tools are likely to reflect the language of the particular scale used, and not the language of the study participants. International guidelines and standards released in 2022 for the study of death and recalled experiences of death address this issue by providing frameworks for the lexicon that is integral to these fields of research, definitions, and also discussing novel scales that can be used in future research [5]. Within this statement by Parnia et al., the authors highlight that as near-death experiences were not formally defined, much of the early evidence base is comprised by research on human experiences that are significantly heterogeneous, and often unrelated to eachother or death [5]. Many studies are often confounded as recalled experiences of death, including near-death experiences, may be challenging to discern from other experiences during coma or unconsciousness, such as confusion or delirium, conventional dreams, intensive care unit (ICU) delusions, experiences when entering or emerging from coma, or consciousness during cardiopulmonary resuscitation (CPR) [5]. An overall narrative of recalled experiences of death is also proposed: (1) perceived death and separation from the body, (2) heading to a ‘destination’, (3) reliving the recording of life that is educational, (4) being ‘home’ again, (5) returning back to life, and (6) reported effects after the experience [5]. Accordingly, to build on these guidelines with an updated synthesis of the relevant evidence base, this scoping review aimed to characterise near-death experiences in the setting of cardiac arrest, namely with respect to (1) incidence, (2) predisposing factors, (3) content, and (4) association with long-term outcomes.

## Methods

Methods for this scoping review were adherent to the PRISMA Extension for Scoping Reviews (PRISMA-ScR) reporting guidelines [6].

### Search strategy and selection criteria

The population, intervention, comparator group, and outcome (PICO) framework was used to develop the research question and inclusion criteria. The population group included survivors of cardiac arrest. The intervention was defined as circumstances leading to cardiac arrest. The comparator group comprised patients that did not have near-death experiences, if reported. The outcome was details of near-death experiences. Only prospective studies were included. Publications reporting single-patient case reports, retrospective data, or not reporting observational data (e.g., editorials, perspectives, research letters, or abstracts) were excluded. Studies published in languages other than English were also excluded. PubMed (incorporating MEDLINE) was searched from database inception to 23 July 2023 for studies of any design and in any setting using the search strategy: ("heart arrest"[MeSH Terms] OR ("heart"[All Fields] AND "arrest"[All Fields]) OR "heart arrest"[All Fields] OR ("cardiac"[All Fields] AND "arrest"[All Fields]) OR "cardiac arrest"[All Fields]) AND ("near death experience*"[All Fields] OR "near death experience*"[All Fields]). No publication restrictions were implemented and searches were not limited by language.

### Data extraction and analysis

A single reviewer screened titles and abstracts and reviewed full-texts. Multiple reviewers extracted data using a standard extraction form. Extracted data included study design and setting, population characteristics, intervention characteristics, quantitative and qualitative outcomes, methodological quality information, and other information relevant to the review questions. Data were synthesised in narrative and tabular formats. Qualitative data relevant to the study outcomes were coded and synthesised according to thematic commonality. Relevant quantitative data were summarised to determine effect sizes across the included studies. Meta-analysis was precluded due to heterogeneity in reported data across the included studies.

## Results

### Study characteristics

**Fig. 1:**
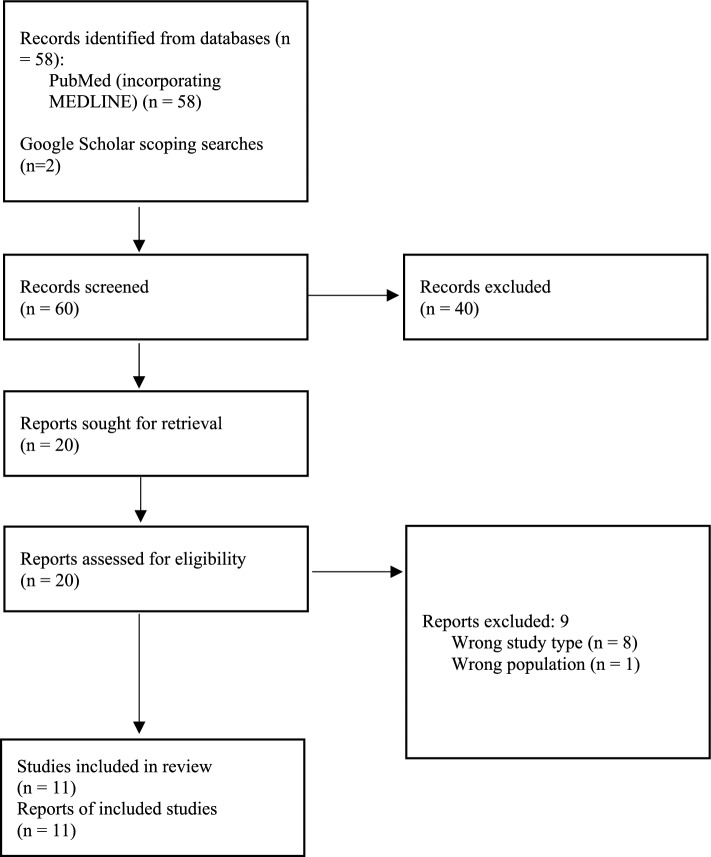
Study selection

The search identified a total of 60 records, from which 20 full-text articles were retrieved and 11 of these studies were included in the scoping review (Fig. 1). A complete list of the studies excluded upon full-text review, in addition to justification for their exclusion, can be found in Additional file 1. Characteristics of the included studies are presented in Table 1. Three studies were conducted in Slovenia [7–9], two in the USA [10, 11], one in the UK [12], one in the Netherlands [13], one in Austria [14], one in Sweden [15], and two international studies [16, 17].

**Table 1:** Study characteristics

First author	Year	Design	Country	Sample Size*	Proportion with NDE (%)	Female (%)	Age	Total follow-up
Greyson[10]	2003	Comparative cohort study	USA	116	10%**			
Klemenc-Ketis[35]	2010	Cohort study	Slovenia	52	11 (21.2%)	10 (19.2)	Median 53.1 (SD: 14.5)	Nil
Klemenc-Ketis[36]	2011	Cohort study	Slovenia	52	11 (21.2%)	10 (19.2%)	Median 53.1 (SD: 14.5)	Nil
Klemenc-Ketis[7]	2013	Cohort study	Slovenia	37	7 (18.9%)	8 (21.6%)	Mean 54 (SD: 13.1)	6 months
Parnia[12]	2001	Cohort study	UK	63	4 (6.3%)**			Nil
Parnia[16]	2014	Cohort study	International	140	9 (6.4%)**	45 (33%)	Mean 64 (SD: 13)	3 months to 1 year
Parnia[17]	2023	Cohort	International	28	11 (39.3%)**	4 (14.3%)	Mean 63.6 (SD: 13.7)	Discharge or death
Schwaninger[11]	2002	Cohort study	USA	30	7 (23%)**	15 (50%)	Mean 60 (range: 23–86)	6 months
Sterz[14]	2023	Cohort study	Austria	126	20 (15.9%)	9 (45%)	Mean 58.7	553 days
Van Lommel[2]	2001	Cohort study	Netherlands	344	62 (18%)	93 (27%)	Mean 62.2 (SD: 12.2)	2 years
Zingmark[15]	2022	Cohort study	Sweden	30	5 (16.7%)	0 (%)		10 weeks

^**^Deemed an ‘experiencer’ with a score of 7 or greater on Greyson’s near-death experience scale[3]

### Incidence of near-death experiences

The reported incidence of near-death experiences in the included studies varied from 6.3% to 39.3% [9, 17]. Aspects of variation in incidence may be due to methodological variations in the definition of near-death experience and differences in ascertainment methods. For example, in one study, which found a incidence of 18%, near-death experience was defined based upon the reported memory of impression of having experienced an altered state of consciousness, rather than a validated scale [13]. The inclusion of in-hospital cardiac arrest patients (reported near-death experience incidences of 10% [10], 6.3% [12], 6.4% [16], 23% [11], 39.3% [17], 15.9% [14], 16.7% [15], and 18%[13]) as opposed to only out-of-hospital cardiac arrest (reported near-death experience incidences 21.2% [9], 21.2% [8], 18.9%[7]), may contribute to variation. All included studies involved interviews. Systematic differences in individual agreement to participate in interviews may introduce a participation bias. One study that reported that no individuals declined to participate found an incidence of 21.2% [9]. A study that used brief screening interviews to identify experiencers, prior to the full interview found an incidence of 10%.[10], The multi-centered AWARE-II study undertook a detailed process to identify experiencers, then reported an incidence of 39.3% in those interviewed [17].

### Factors associated with near-death experiences

Individual variables in terms of patient characteristics demonstrated statistically significant association with a propensity for near-death experiences. Four studies described possible associations between individual factors and the likelihood of having a near-death experience [8, 9, 12, 13]. However it is important to note that these studies were insufficiently powered to firmly draw conclusions, with multiple major methodological issues and potential sources of bias and confounding present. Accordingly, the literature to date was seen as being insufficient to address the review question relating to factors associated with near-death experiences. One study of 344 patients found that certain demographic and disease characteristics varied between those with a near-death experience and those who did not. Namely, those with a near-death experience were more likely to be under the age of 60 (52% vs 34%, P = 0.011), have a first myocardial infarction (97% vs 84%, P = 0.013), or have had a previous near-death experience (10% vs 3%, P = 0.035) [13]. In a study of 52 patients that focussed on electrocardiogram characteristics, those with pulseless electrical activity had significantly more near-death experiences (P = 0.003), whereas patients with ventricular fibrillation had fewer near-death experiences (P = 0.006) [8]. Likely in the same cohort of patients, a subsequent study that focussed on laboratory parameters found higher initial end-tidal partial pressure of carbon dioxide (5.7 vs 4.4, P < 0.01), and higher arterial partial pressure of carbon dioxide (6.6 vs 5.3, P = 0.041) were associated with an increased likelihood of a near-death experience [9]. A higher score on the near-death experience scale was associated with higher potassium (P = 0.026), but not an increased likelihood of meeting the threshold for a near-death experience. One study that was significantly limited by sample size (four individuals with near-death experiences) did not perform statistical comparisons; however found the mean arterial partial oxygen pressure in the near-death experience group was double that of the control group [12]. In this small study the mean arterial partial pressure of carbon dioxide and serum potassium were similar between groups. The AWARE-II study found several EEG features and biomarkers that are associated with consciousness during cardiac arrest or cardiopulmonary resuscitation [17].

### Content of near-death experiences

The language used within the included studies to characterise the content of near-death experiences reflected the language of the scale used, and not the true language used by the individual study participants. Of the included studies, 9 used the Greyson near-death experience scale[18]. The individual components of the scale that were described most frequently in those who had a near-death experience were presented in three studies. In the largest of these studies, the most commonly endorsed experience were a feeling of peace (85%), seeing/feeling surrounded by light (70%), and a sense of being outside of the physical body (70%) [10]. Similarly, a substantially smaller study described that in those who had a near-death experience a sensation of peace was most common (100%), followed by an external visual or auditory awareness experience (90%), and experiences relating to deceased family members and an entity that was warm, loving and luminous (72%) [11]. In a study that included only four individuals with near-death experiences, the most commonly reported features were coming to a point/border of no return (100%), feelings of peace (75%), feelings of joy (75%), and seeing a bright light (75%) [12]. In one study the results of those who met the criteria for a near-death experience were not presented separately to those who did not fulfil the criteria. Across the entire cohort the most commonly endorsed features were feeling that everything was happening faster/slower than usual (27% of all respondents), a feeling of peace or pleasantness (22%), a feeling that the senses were more vivid than usual (13%), and a feeling of being separate from one's body (13%) [16]. The included study that did not use the Greyson near-death experience scale described the most common experiences to be positive emotions (56%), awareness of being dead (50%), and meeting with deceased persons (32%) [13]. The AWARE-II study extensively characterised the near death experiences, notably discerning five thematic categories: emergence from coma during CPR, emergence from coma in the post-resuscitation period, delusional misattribution of ongoing medical events, recall of experience of death, and dream-like experiences [17]. In the ‘experiencers’ within one study, content comprised meeting with deceased relatives, an external visual or auditory awareness episode, and being sucked into a colourful tunnel [14]. Another study characterised four themes through analysis of interviews via a phenomenological hermeneutical method: being on the other side in another dimension, having a real experience, being in a non-physical condition without their body, and comparing views of life and death before and after the near-death experiences [15].

### Long-term outcomes after near-death experiences

Three studies presented results on differences between patients who had near-death experiences and those who did not after a period of follow-up. Only one of these studies described a difference with respect to medical outcomes, namely that in a non-controlled analysis, revived cardiac arrest patients with a near-death experience were more likely to have died (i.e. experience all-cause mortality) within 30 days than revived cardiac arrest patients who did not have a near-death experience (21% vs 9%, P = 0.008) [13]. This study also described that at a 2-year follow-up interview and survey there were positive differences for the near-death experience group (compared to a non-near-death experience comparator group) on the life-change inventory questionnaire in social attitude, religious attitude, attitude towards death, interest in the meaning of life, and self-understanding. At an 8-year follow-up, although the statistical significance of differences was not presented, life-change inventory questionnaires suggested that positive changes in patients with near-death experiences remained. Similar positive changes in attitude were also found in the other studies. In one study, based on a 6-month follow-up survey, differences between those that experienced near-death experience and those who did not included changes in religious attitudes, changes in global attitudes, perceptions of understanding of one’s own life, attitude to other people, and changes in social practices [11]. Those that experienced near-death experience were also more likely to describe external visual or auditory awareness experiences, and a sense of a surrounding energy force. In the third study, at 6-months, compared to non-near-death experience experiencers, there were significant differences in tolerance for others, self-understanding, nature appreciation, sense of inner-meaning to life, and concern regarding social justice issues [7].

## Discussion

This scoping review found that in prospective studies that have investigated the phenomenon, near-death experiences may occur in as frequent as over one-third of patients with cardiac arrest. It was also observed that lasting effects may follow these events, such as a positive life attitude change and social benefits, but also a potentially increased likelihood of mortality in the shorter term (which was reported by one landmark study in this space). The comorbidities and reasons for cardiac arrest were heterogeneously reported within the synthesised evidence base, and several patient characteristic variables across biopsychosocial domains demonstrated significant association with an increased likelihood of having near-death experiences during cardiac arrest. Further, it was also observed that the reported content of near-death experiences within the literature often reflected the language of the specific questionnaire that was used, rather than the true language reported by individual participants within the studies. However, the most commonly described components of a near-death experience include feelings of peace, a sensation of light, and external visual or auditory awareness. It must also be noted that there were some methodological concerns within many of the studies, which may confound the conclusions drawn.

The 2022 guidelines and standards for the study of death and recalled experiences of death by Parnia et al. presents the most comprehensive synthesis of knowledge relating to near-death experiences to date [5]. Over 50 themes have been previously associated with the content of near-death experiences, which can be grouped under the major headings of separation, heading to a ‘destination’, reliving the recording of my life: actions and intentions matter, ‘home’ again, the return, and reported effects after the experience [5]. Conceptual distinctions are made within this piece, such as proposing the use of ‘recalled experience of death’ (a specific cognitive experience occurring during a period of loss of consciousness in relation to a life-threatening event, including cardiac arrest) in place of ‘near-death experience’, mainly as the phrase was previously misused to describe experiences not relating to death in the past literature [5]. Other publications have synthesised the literature on CPR-related consciousness and cognitive activity, which although still developing is growing in its frequency of being reported, and have been referenced in recent international CPR guidelines. [16, 19, 20] However, these related concepts and phenomena were was outside the scope of the present study, and inclusion criteria specifying the presence of cardiac arrest alongside the phraseology of ‘near-death experience’ was used to capture relevant prospective studies in the past evidence base. However, the new terminology should be used going forward as per the Parnia et al. expert consensus [5].

Recent and robust 2023 evidence suggests some biomarkers and electroencephalogram findings may be associated with near-death experiences [17]. Increased gamma oscillations and functional connectivity have been observed in electroencephalograms in the first five minutes after cardiac arrest, supporting patient descriptions of heightened consciousness and lucid thought processes; acting as a possible physiological correlate to the occurrence of human recalled experiences of death [21–24]. Within a study of electroencephalograms and electrocardiograms of four comatose dying patients by Xu et al., surges of functional connectivity were observed across several frequency bands [22]. However, the processes underlying the *enduring* effects of a near-death experience, particularly changes across multiple psychological and social domains for up to eight years following the near-death experience[13], remain unclear. However, enduring psychological changes following transient experiences have been described in association with some pharmaceuticals, which may be relevant [5]. Most notably, ketamine may have an antidepressant effect beyond the time which it would typically be considered pharmacologically active [25]. Ketamine, and to a lesser degree some serotonergic psychedelics and deliriant alkaloids, have been reported in the existing literature to result in altered conscious states that are similar to near-death experiences [26]. Post-traumatic growth may contribute to the lasting effects of near-death experiences, and have been previously discussed in this setting [27]. In addition to ketamine [26], N,N-Dimethyltryptamine (DMT) [28], meditation [29, 30], REM sleep intrusion [31], and syncope[32], are among variables that have been linked with the induction of experiences similar to those within near-death experiences [5].

It has been proposed that a near-death experience can be considered as a probe to study aspects of consciousness. This is similar to ICU-delirium, and CPR-related consciousness [5,19,33]. Although definitions vary, full consciousness is generally considered to require both wakefulness (*level* of consciousness) and awareness (*content* of consciousness), which can be impaired independently (e.g. in a vegetative state, wakefulness is preserved while awareness is impaired) [34]. Near-death experiences have been postulated to represent a type of *disconnected* consciousness, which is a state of reduced wakefulness, reduced awareness of the environment, and preserved awareness of the self [1]. If an individual has a subjective experience during a cardiac arrest, while unrousable and unaware of the external environment, this event fulfils the conditions for disconnected consciousness. Accordingly, the future investigation of near-death experiences may provide unique insights into conscious states, particularly the processes required to generate and maintain disconnected consciousness.

This study has multiple limitations. Prominent near-death experience questionnaires used within the past literature and included studies are validated and have utility [3, 4], but also have limitations. In particular, as the tools categorise themes using certain language, the results from studies that use these tools, and any synthesis of these data such as the present scoping review, reflects the language of the scales used, and not necessarily the exact language used by the study participants when describing their near-death experiences. This must be acknowledged when interpreting findings from any study investigating near-death experiences, both in the past and future literature. Two of the included studies were associated with the same patient cohort and conducted by the same investigators [35, 36], increasing the potential for bias from these data. The nature of the included studies is that they rely upon self-reporting of near-death experiences and it is possible that concerns regarding stigma associated with mental illness may limit the disclosure of some participants. Again, inherent to the nature of the subject matter, recall bias may influence study results. The relatively few studies included increases the risk for bias. Further, all of the included studies used near-death experience scales that did not permit the identification of distressing or negative near-death experiences. Future prospective research should seek to overcome this limitation through the use of novel near-death experience scales, such as the Near-Death Experience Content (NDE-C) scale [37], that do allow for differentiation of negative or distressing near-death experiences. It is important to note that the studies investigating factors associated with near-death experiences were insufficiently powered to reliably derive conclusions, with multiple methodological issues and potential sources of bias present. It is also noteworthy that multiple studies, of the few studies included, were conducted by the same investigators.

## Conclusions

Near-death experiences may occur in as frequent as over one-third of patients with cardiac arrest. Enduring effects may follow these events, such as a positive life attitude change and social benefits, but there may also be a potentially increased likelihood of mortality in the shorter term. Some individual variables in terms of patient characteristics demonstrated association with an increased likelihood of having near-death experiences during cardiac arrest, however many of the underlying mechanisms remain unclear. There are some methodological concerns within many of the prospective studies in this literature, which may confound the conclusions drawn. Longer-term outcomes may have been biased by clinical characteristics and comorbidities, rather than near-death experiences, and this should remain a pertinent consideration. Future research should examine the biological, pragmatic, theoretical, and philosophical aspects of near-death experiences, adhering to recent international guidelines in this space [5]. Further investigation of the described association with an increased risk of 30-day mortality on non-controlled analysis is required, as this may identify a subgroup of patients who would benefit from further intervention. Described effects on mental health outcomes and medication adherence may also have practical implications. Further, deeper investigation into near-death experiences may provide insights into the processes relating to generating and maintaining disconnected consciousness.

### Supplementary Information

Below is the link to the electronic supplementary material.

Supplementary file 1 (DOCX 164 KB)
